# Seed train optimization for suspension cell culture

**DOI:** 10.1186/1753-6561-7-S6-P9

**Published:** 2013-12-04

**Authors:** Tanja Hernández Rodríguez, Ralf Pörtner, Björn Frahm

**Affiliations:** 1 Department of Mathematics, Bielefeld University, Bielefeld, D-33615, Germany; 2 Institute of Bioprocess and Biosystems Engineering, Hamburg University of Technology, Hamburg, D-21073, Germany; 3 Biotechnology & Bioprocess Engineering, Ostwestfalen-Lippe University of Applied Sciences, Lemgo, D-32657, Germany

## Fields of application

Fields of application are the production of biopharmaceuticals (antibodies, proteins for diagnostic and therapeutic purposes) based on suspension cell culture and cultivation scales and -systems of any kind.

## Introduction

The purpose of a seed train is the generation of an adequate number of cells for the inoculation of a production bioreactor. This is time- and cost-intensive: From volumes used for cell thawing or cell line maintenance the cell number has to be increased. The cells are usually run through many cultivation systems which become larger with each passage (e.g. T-flasks, roller bottles or shake flasks, small scale bioreactor systems and subsequently larger bioreactors. Single-use systems may be applied and systems which are inoculated at a partly filled state and culture volume is increased afterwards by medium addition). The production bioreactor is inoculated out of the largest seed train scale.

## Motivation

A seed train offers space for optimization, e.g. choice of optimal points in time for cell passaging from one scale into the larger one. Furthermore choice of inoculation cell density as well as culture volume at inoculation in bioreactor scales (when inoculation volume is below maximum working volume). When designing a new seed train, the volumes of the cultivation scales may also be open for optimal choice.

## Results

### Tool structure

A seed train structure has been programmed in Matlab^®^. The implemented model calculates cell growth, cell death, uptake of substrates and production of metabolites. The tool is suitable for different cell lines via entering corresponding model parameters, medium and seed train information. Seed train optimization is possible regarding cell passaging at optimal Space-Time-Yield (STY) or other optimization criteria [1].

### Application example for CHO cell line

**Figure 1:**
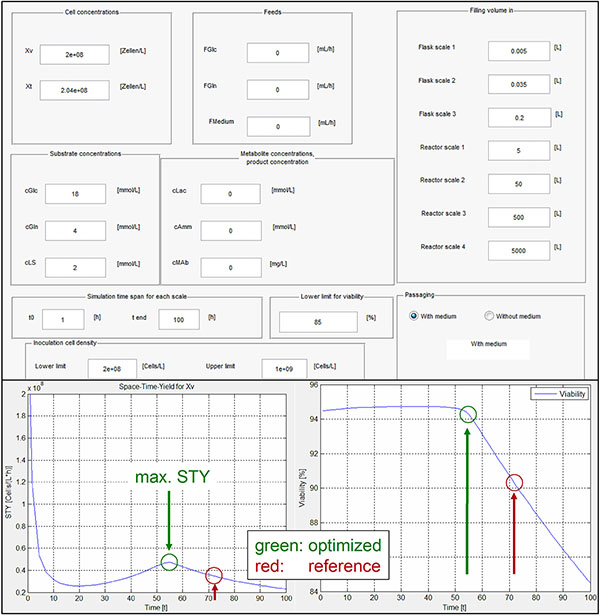
**One input mask of the seed train starting conditions as an example for the tool's user interface and courses of Space-Time-Yield (STY) and viability over time during growth for flask scale 2**.

Based on three cultivations, cell line model parameters have been determined using the simplex algorithm by Nelder and Mead. The whole seed train is modeled for cell passaging at fixed time intervals (current method, reference) and cell passaging at optimal points in time (optimized method). For this, the tool calculates Space-Time-Yield-(STY)-courses for every scale and selects the optima. As examples, Figure 1 shows an input mask of the seed train starting conditions as well as the courses of STY and viability over time during growth for flask scale 2:

Figure 1 indicates that the reference method passages the cells in T-flasks and roller bottles when Space-Time-Yield (STY) is already decreasing and viability dropping which is too late (beginning of stationary phase, not presented).

The whole optimized seed train is calculated including optimal points in time for cell passaging and optimal inoculation volumes and -densities in reactor scales. Table 1 gives an example of an output screen.

**Table 1:**
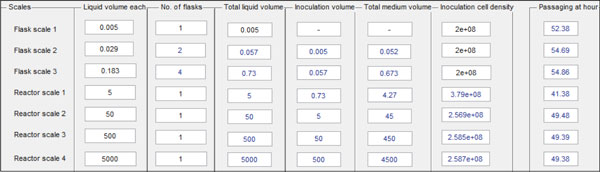
**Output screen example displaying the whole seed train including inoculation of production bioreactor (reactor scale 4, 5,000 L)**.

In this example, time saving until inoculation of a 5,000 L production bioreactor is 108 hours. When the averages of point in time of optimal Space-Time-Yield (STY) and point in time of growth rate decreased to 90% are taken, time saving is 114 hours. This method also offers a 'safety' time span between cell passaging and beginning of stationary phase.

## Conclusions

The tool improves seed train understanding and allows seed train design and optimization. Time savings as well as increased viabilities for passaging are possible. The tool has also been tested using a known and manually optimized seed train. Without such time consuming lab work, the tool has delivered the same optimized seed train only based on data of two batches [2].
